# Craniorachischisis totalis

**DOI:** 10.11604/pamj.2023.44.24.35962

**Published:** 2023-01-12

**Authors:** Suyash Yashwant Ambatkar, Megha Dipak Rudey

**Affiliations:** 1 Department of Orthopaedics, Datta Meghe Institute of Medical Sciences (DU), Sawangi, Wardha, Maharashtra, India,; 2 Department of Kaumarbhritya, Mahatma Gandhi Ayurveda College, Hospital and Research Centre, Salod (H), Datta Meghe Institute of Medical Sciences (DU), Sawangi, Wardha, India

**Keywords:** Craniorachischisis, anencephaly, spina bifida, rachischisis

## Image in medicine

Craniorachischisis totalis is a lethal, non-syndromic anomaly. This anomaly is a rare condition seen in neural tube defects, characterized by anencephaly (absence of brain and cranial vault and lack of skin covering), and bony defect of the cervical spine (without covering of meninges on neural tissue). The prevalence of craniorachischisis in Europe is 0.1 to 10.7 per, 10000 live births. Many of the fetuses are born either stillborn or result in termination of pregnancy if diagnosed prenatally. A study on non-genetic risk factors in craniorachischisis totalis is hard-hitting because of its rarity. A recent chromosomal study showed mutation of the SCRIB and CELSR1 genes leads to chraniorachischisis totalis. Adequate supplementation of folic acid before conceiving and in the antenatal period can lead to the uppermost hand for the prevention of craniorachischisis up to 75%. Antenatal diagnosis and termination of pregnancy are the main reasons that have helped craniorachischisis become uncommon. We report a clinical image of the male fetus with craniorachischisis totalis. The gestational age of the fetus was 14 weeks, with the crown to rump length of 120 millimetres. This fetus has spina bifida without a brain and covering of meninges, followed by spontaneous abortion in the institutional hospital of Maharashtra state in Central India. The obstetric history was not available. This condition is not compatible with life, so the fetus undergoes spontaneous abortion in the initial few weeks of gestation itself.

**Figure 1:**
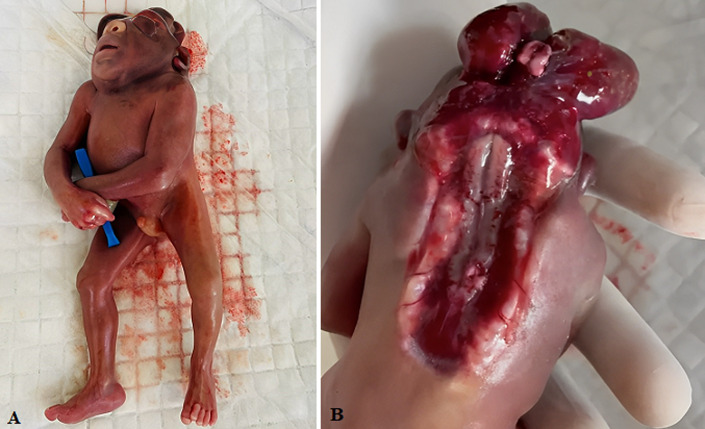
A) total craniorachischisis (absence of brain and cranial vault and lack of skin covering) in the fetus of 14 weeks of gestational age; B) absence of meningeal covering

